# The Effect of Different Surface Mechanical Attrition Treatment Time on the Fretting Wear Properties of TC4 Alloy in Artificial Seawater

**DOI:** 10.3390/ma19010123

**Published:** 2025-12-30

**Authors:** Xiaoxiao Luan, Sujuan Yu, Zhenlin Liu, Shaohua Yin, Feng Xu, Xiaofeng Zhang, Long Xin

**Affiliations:** 1 Department of Medical Engineering, Peking University Third Hospital, Beijing 100191, China; bysy_ysj@126.com (S.Y.); 1263188462@bjmu.edu.cn (Z.L.); ykdyinsh@163.com (S.Y.); xusteven@sohu.com (F.X.); 2 National Center for Materials Service Safety, University of Science and Technology Beijing, Beijing 100083, China; zhangxiaofeng@ustb.edu.cn

**Keywords:** TC4 alloy, SMAT, microstructure, fretting wear, grain size

## Abstract

The TC4 alloy is widely used in aerospace and marine engineering due to its excellent mechanical properties and corrosion resistance. However, titanium alloys often face fretting wear problems during use, which affect their long-term stability and service life. This study investigates the effects of surface mechanical attrition treatment (SMAT) time on the surface morphology, microstructure, stress distribution, and fretting wear properties of TC4 alloy. Characterization was performed using white light interferometry, EBSD, SEM, XRD, and microhardness measurements. The results show that SMAT significantly changes the surface and wear properties of TC4 alloy. With the increase in SMAT time from 0 to 240 min, the surface roughness (Ra), hardness, deformation depth, and stress gradually increase while the grain size decreases. After 240 min of SMAT, the TC4 alloy exhibited optimal fretting wear resistance, achieving a wear depth of 14.27 μm, a wear volume of 2.48 × 10^6^ μm^3^, and a wear rate of 1.24 × 10^3^ μm^3^/s. This represents a significant improvement, corresponding to an approximate 32.8% reduction in wear depth and a ~48% reduction in both wear volume and wear rate compared to the untreated sample.

## 1. Introduction

Titanium and titanium alloys have been widely used in aerospace [1], biomedical [2], and marine engineering [3] due to their excellent specific strength, corrosion resistance, and biocompatibility. Among them, TC4 alloy (Ti-6Al-4V), as a typical α + β dual-phase titanium alloy, has become one of the most widely used titanium alloy materials in industrial applications due to its good comprehensive properties [4,5]. Especially in the marine environment, its corrosion resistance and high-strength characteristics make it an ideal material choice [6,7]. However, TC4 alloy still faces some challenges in practical applications, such as low surface hardness, insufficient wear resistance and fatigue performance in complex stress environments [8,9]. These problems limit its further application under extreme conditions. Therefore, how to improve the surface properties of TC4 alloy by surface modification technology has become one of the research hotspots in the field of material science and engineering in recent years.

Fretting wear [10], characterized by small-amplitude oscillatory motion between contacting surfaces, is a critical degradation mode in assembled marine components such as bolted joints, bearing housings, and blade roots. For titanium alloys like TC4 in seawater environments, the fretting process is particularly complex due to the interplay of mechanical wear and electrochemical corrosion, a synergy often termed tribocorrosion. The inherent susceptibility of titanium alloys to adhesive wear, combined with the cyclic nature of fretting, can lead to accelerated material removal through mechanisms including abrasion by third-body debris, oxidative wear facilitated by the rupture and reformation of passive oxide films, and delamination caused by subsurface crack initiation and propagation. The presence of a corrosive medium like seawater can further exacerbate material loss by dissolving wear debris, preventing the formation of a protective tribolayer, and promoting corrosion-assisted crack growth. Therefore, enhancing the resistance of TC4 alloy to this combined mechanical–chemical damage is paramount for ensuring the longevity and reliability of marine structures.

There have been several studies reporting improved surface hardness and wear resistance by modifying the (i) surface asperities and (ii) microstructure, collectively known as surface integrity. Tailoring the latter is possible through thermo-chemical treatments like nitriding, carburizing, ion implantation, and deposition of hard coatings apart from employing surface and Sub-Surface Plastic Deformation (SSPD) processes [11]. Surface mechanical attrition treatment (SMAT) is a surface modification technology that induces nanocrystalline structures on the surface of materials by mechanical impact [12,13]. By introducing severe plastic deformation on the surface of the material, SMAT refines the surface grains to nanoscale, and introduces residual compressive stress and lattice distortion, thereby significantly improving the surface hardness, wear resistance, and fatigue resistance of the material [14]. In recent years, SMAT technology has been widely studied in a variety of metal materials, such as stainless steel, aluminum alloy, and magnesium alloy; however, research on its application to TC4 alloy has been relatively limited [15,16]. Through SMAT, the surface microstructure of titanium alloy changes significantly, forming nanoscale grains, which is considered to be a key factor to improve its wear resistance [17]. Studies have shown that SMAT can effectively improve the surface hardness and surface roughness of the material, and significantly improve the friction and wear properties of titanium alloys through grain refinement, optimization of residual stress, and strengthening of surface structure [12]. However, there is still a lack of systematic research on the effects of different SMAT times on the surface morphology, roughness, and crystal structure of TC4 alloy. In addition, the influence mechanism of SMAT on the fretting wear properties of TC4 alloy also needs to be further discussed. Therefore, it is not only of great scientific significance to systematically study the effect of SMAT time on the surface and subsurface properties of TC4 alloy, but also provides theoretical support for expanding the application field of TC4 alloy.

With the extension of SMAT time, the influence of surface microstructure evolution on material properties becomes more and more obvious [18]. Under different SMAT times, the surface roughness, grain size, interface structure, and wear behavior of TC4 titanium alloy will change. In addition, SMAT can also lead to changes in the residual stress distribution of the surface layer [19,20], which in turn affects the overall mechanical properties and wear characteristics of the material. These changes not only help us to understand the influence of different times on TC4 alloy, but also provide a theoretical basis for further optimizing its performance in practical applications.

TC4 titanium alloy is widely used in marine engineering equipment, such as sea water pumps, fasteners, propellers, and other key components [21,22]. In the working process of these components, they face harsh environments such as high friction and high load. Therefore, the wear resistance and corrosion resistance of the material surface are particularly important. In order to solve these problems, SMAT has been proposed as an effective modification method to improve the wear resistance and surface hardness of TC4 alloy [23,24]. By adjusting the SMAT time, the microstructure of the surface layer can be accurately controlled, thereby optimizing the performance of the alloy [23], especially under different wear conditions. However, there is still a lack of systematic research on the effect of different SMAT times on the surface morphology gradient evolution, stress distribution, and local misorientation of TC4 alloy.

The research in this paper mainly focuses on the surface morphology, microstructure evolution, stress distribution, and its influence on fretting wear properties of TC4 titanium alloy under different SMAT times. By systematically analyzing the surface and subsurface microstructure changes in TC4 alloy under different SMAT times, it provides new experimental data for further understanding the optimization mechanism of SMAT on the surface properties of titanium alloy. In addition, this paper also discusses the effect of SMAT on the overall performance of TC4 alloy, which provides a theoretical basis for optimizing SMAT process parameters and further improving the wear resistance and mechanical properties of TC4 alloy.

## 2. Materials and Methods

**Figure 1:**
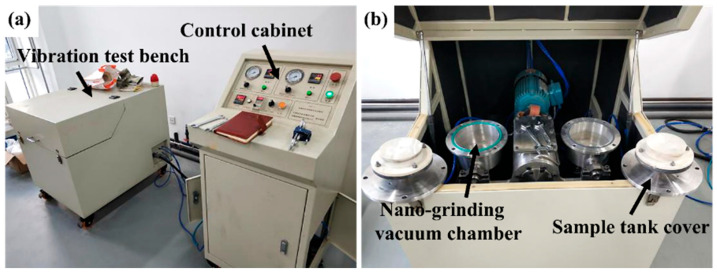
SNC-1 surface nanocrystallization testing machine. (**a**) The whole picture of the testing machine, (**b**) the interior of the vibration test bench.

**Figure 2:**
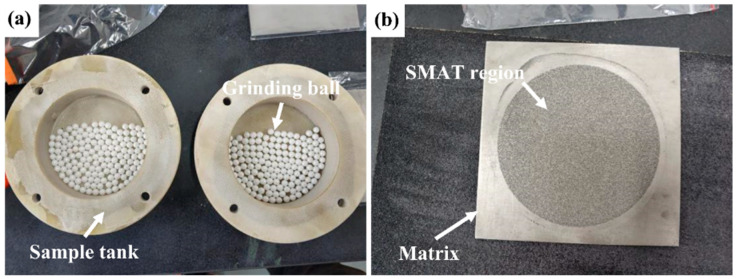
Sample tank and SMAT sample. (**a**) Sample tank with grinding balls, (**b**) sheet specimen after SMAT.

The test used annealed TC4 titanium alloy (international brand Ti-6Al-4V), and its chemical composition and microstructure have been introduced before [25]. The test material was annealed TC4 titanium alloy (Ti-6Al-4V, Grade 5). The annealing heat treatment was performed at 700 °C for 2 h, followed by furnace cooling. This process results in a bimodal microstructure consisting of equiaxed primary α phase and transformed β phase, providing a baseline microstructure with a Vickers hardness of approximately 364 HV. The surface mechanical attrition treatment (SMAT) of TC4 alloy sheet was carried out by using yttrium-stabilized TZP zirconia ceramic ball. The equipment used in SMAT is SNC-1 surface nanocrystallization testing machine (Chengdu, China), as shown in Figure 1. The testing machine includes two parts: control cabinet and vibration test bench. Two surface nano-grinding vacuum chambers are installed symmetrically in the vibration test bench. The surface mechanical grinding plate is placed between the customized fiber-reinforced plastic/polymer (FRP) sample tank and the sample tank cover. The FRP sample tank and the upper cover of the grinding chamber are bolted. The zirconia grinding ball is placed in the sample tank, and the plate sample above the sample tank is hit with the vibration of the shaking table, as shown in Figure 2. The equipment parameters of the metal surface nanocrystallization testing machine are listed in Table 1.

The TC4 alloy plate was cut into a block plate of 100 mm × 100 mm × 6 mm. After being polished by a grinder, the surface roughness (Ra) of the plate reached 0.396 μm, and then was ultrasonically cleaned with acetone for 5 min and dried for later use. In this experiment, the SMAT of TC4 titanium alloy was carried out by using a combination of zirconia ceramic balls with a diameter of 5 mm and a sample tank made of glass fiber-reinforced plastic. The zirconia ceramic balls are placed at the bottom of the sample tank container, the number is about 2/3 of the bottom, and the treated sample is fixed at the top of the container. The vibration frequency of the shaking table is set to 50 Hz, and the single treatment time is 30 min (a single operation cannot exceed 30 min, and the sample tank needs to be cooled). The sample is removed every 60 min for cleaning to remove the transfer material on the surface, and the total treatment time is 60 min, 120 min, 180 min, and 240 min. The samples without SMAT were recorded as SMAT 0 min, and the samples with SMAT for 60 min to 240 min were recorded as SMAT 60 min, SMAT 120 min, SMAT 180 min, and SMAT 240 min, respectively. In the process of surface mechanical grinding, the vibration table in the surface nanocrystallization equipment vibrates up and down at a set frequency, driving the vacuum grinding chamber container assembled on it, so as to drive the zirconia grinding ball in the sample tank to randomly impact the surface of the treated material from all directions. With the repeated impact of the ball, the surface of the sample continuously produces strong plastic deformation (high strain rate), and the surface of the sample realizes gradient nanocrystallization. SMAT parameters include the vibration frequency = 50 Hz (as stated), amplitude = 2 mm, and the total treatment times were 60, 120, 180, and 240 min, with intermediate cooling and cleaning every 60 min.

In order to study the wear performance under different SMAT times, 30 mm × 30 mm × 6 mm blocks were cut on the plate after SMAT for 60 min, 120 min, 180 min, 240 min, and the original base metal, respectively, as fretting wear samples. Prior to testing, all sample surfaces were ground with SiC sandpapers and polished using diamond paste to achieve a surface roughness (Ra, arithmetical mean deviation) of approximately 0.05 μm. The fretting wear test was carried out on the high frequency reciprocating module of MFT-5000 multifunctional friction and wear tester (Rtec-Instruments, San Jose, CA, USA), employing a ball-on-plate contact configuration. An Al_2_O_3_ ceramic ball (diameter: 5.953 mm) served as the counter-body material. The test was carried out in simulated seawater. In the fretting wear test, the loading force was 2 N, the fretting frequency was 10 Hz, the amplitude was 150 μm, and the number of cycles was 10,000. Prior to fretting wear testing, all SMAT-treated samples were lightly ground and polished using diamond paste to standardize the surface roughness (Ra ≈ 0.05 µm) for consistent tribological contact. This process removed a superficial layer of less than 2 µm, which is negligible compared to the multi-ten-micrometer depth of the SMAT-induced gradient microstructure and hardened layer, thereby ensuring the tested surface properties were representative of the SMAT-treated state.

The fretting wear test was carried out in simulated seawater. The composition of the artificial seawater is detailed in Table 2. The solution’s pH was adjusted to 8.2 to simulate the natural seawater environment.

After the test, the specimen was placed in alcohol for 10 min of ultrasonic cleaning, and after drying, the characterization analysis was carried out. The surface roughness, wear morphology, wear volume, and wear depth of TC4 alloy before and after SMAT and different time of SMAT were analyzed by white light interferometer. The microhardness of the cross-section of TC4 titanium alloy after SMAT was measured by Vickers hardness tester through static indentation method, applying a load of 100 g and a dwell time of 10 s. The grain morphology and size distribution of TC4 alloy after SMAT were studied by electron backscatter diffraction (EBSD, Oxford Instruments, Abingdon, UK). The EBSD sample preparation process is consistent with the metallographic sample preparation process in the early stage [25]. Cross-sectional specimens after SMAT were mounted, ground, and polished to a mirror finish. Stress-relief polishing was then conducted on a vibratory polisher at a frequency of 90 Hz and an amplitude of 50%, using a 0.05 μm colloidal silica suspension for 4 h. After polishing, the samples were ultrasonically cleaned in anhydrous ethanol for 15 min and air-dried. X-ray diffractometer (XRD, Bruker D8 ADVANCE, Mannheim, Germany) was used to analyze the composition of the surface of TC4 alloy without SMAT and after SMAT for different times and the cross-sections of TC4 alloy at different depths from the SMAT surface after SMAT. The diffraction data were collected across a 2θ range of 25–85°, employing a step width of 0.01° and a counting time of 5 s per step. The working voltage was 45 kV. The surface wear morphology of TC4 alloy after SMAT was studied by scanning electron microscopy (SEM, Zeiss, Oberkochen, Germany) at an accelerating voltage of 15 kV and a working distance of 9.7 mm.

It is important to note that the Kernel Average Misorientation (KAM) parameter provides a qualitative assessment of local plastic strain and the density of geometrically necessary dislocations (GNDs). While these factors are intrinsically linked to the development of residual stresses, KAM analysis does not provide a direct quantitative measurement of macroscopic residual stress, which requires dedicated techniques such as the X-ray diffraction (XRD) sin^2^ψ method.

## 3. Results and Discussion

### 3.1. The Effects of Different SMAT Time on the Surface Morphology, Roughness and Crystal Structure of TC4 Alloy

**Figure 3:**
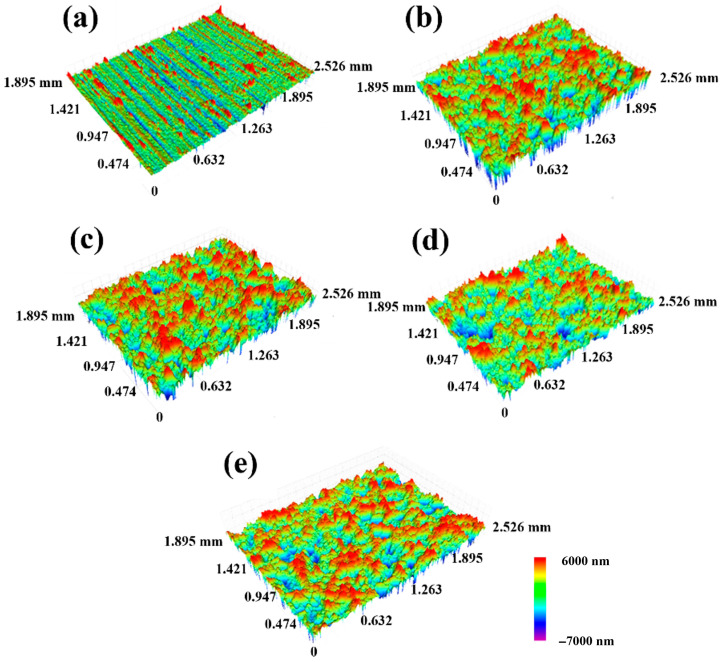
Three-dimensional morphology of TC4 surface at different times of SMAT. (**a**) 0 min, (**b**) 60 min, (**c**) 120 min, (**d**) 180 min, (**e**) 240 min.

The surface morphology of the samples under different SMAT times was optically measured by a white light interference profilometer. Figure 3 shows the three-dimensional surface topography of the sample under different SMAT times, in which the green area represents the vicinity of the datum plane, the red area represents the high point (higher than the datum plane), and the blue area represents the low point (lower than the datum plane). From the diagram, it can be seen that the surface of the sample without SMAT presents parallel and relatively uniform wear marks; on the surface of the sample treated with SMAT for 60 min, there are more peaks (red areas), fewer valleys (blue areas) and lighter colors, indicating that the depth of the valleys is shallow, and the number of deeper valleys is within 5. With the increase in SMAT time to 120 min, the peak of the sample surface decreased significantly, while the valley area increased significantly, and the number of deeper valleys increased to about 20. When the SMAT time was further extended to 180 min and 240 min, the area of peaks on the surface of the sample was further reduced, the area of valleys continued to increase, and the number of valleys increased and showed a continuous trend. Through the comparative analysis of the optical images of the surface morphology of the samples with different SMAT times, it can be found that the original wear marks on the surface of the sample gradually disappear under the impact of the grinding ball. At the same time, with the increase in SMAT time, the area of the peak on the datum plane of the sample gradually decreases, indicating that the datum plane tends to be gentle. The increase in the valley area is attributed to the influence of plastic deformation caused by successive impact on the surface during SMAT and the impact of the broken grinding tank and grinding ball particles on the surface of the sample. These particles fall off after ultrasonic cleaning, leaving more pits.

**Figure 4:**
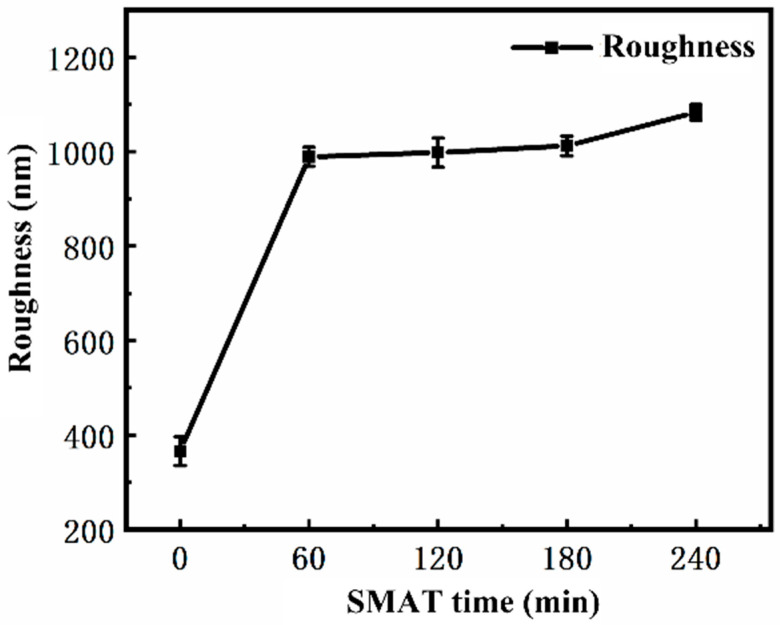
Surface roughness curves after SMAT for different times.

The surface roughness of the samples treated with SMAT for different times was tested by a surface profiler. Five points were randomly selected on the surface of each sample to measure the roughness Ra and take the average value, and the results were retained to an integer. Based on these data, the curve of roughness with SMAT time is drawn, as shown in Figure 4. It can be seen from the diagram that the surface roughness of the sample increases significantly with the increase in SMAT time, and the surface roughness after SMAT is much higher than that of the original sample. This rule is consistent with the change in surface morphology observed in the previous section: although with the increase in SMAT time, the area of surface protrusions decreases, and the part above the datum plane tends to be gentle, the number of pits below the datum plane increases and the area increases. These pits have a more significant effect on surface roughness, resulting in an upward trend in roughness Ra with the increase in SMAT time.

To complement the Ra analysis and provide a more robust description of the three-dimensional surface morphology, the Developed Interfacial Area Ratio (Sdr) was calculated from the topographic data. The Sdr value increased from ~2.5% for the untreated sample to over ~18% after 240 min of SMAT. This significant increase in Sdr quantitatively confirms the transition from a relatively flat, ground surface to a highly textured and complex morphology dominated by pits and valleys, which aligns with the qualitative observations in Figure 3. While Ra describes the vertical deviations, Sdr effectively captures the increase in surface complexity and true contact area, which are critical for tribological behavior.

**Figure 5:**
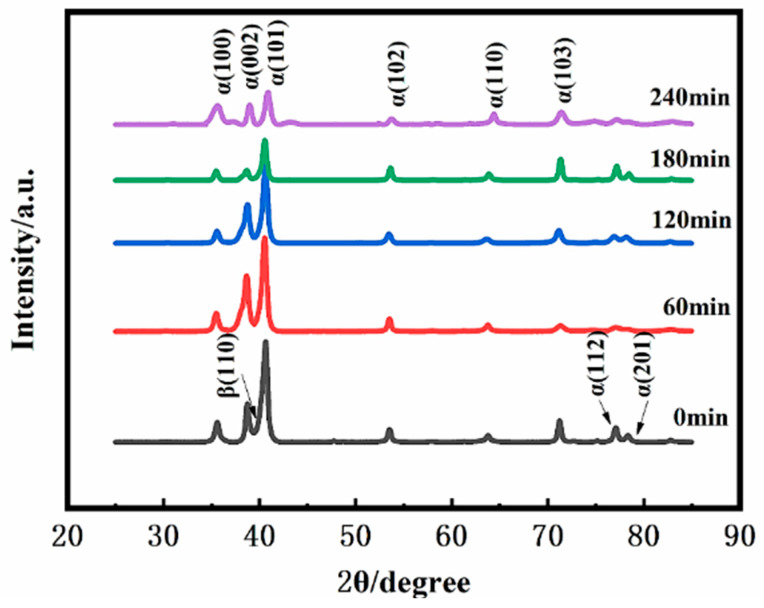
XRD patterns of the sample surface after SMAT for different times.

Figure 5 shows the XRD patterns of the TC4 alloy surface after SMAT for different durations. The primary change observed is in the phase composition. With increasing SMAT time, the diffraction peaks corresponding to the β phase gradually weaken and eventually disappear, indicating a significant reduction in its detectable content, likely due to phase transformation or severe lattice distortion induced by plastic deformation. Concurrently, the diffraction peaks of the α phase exhibit noticeable broadening and a slight increase in intensity. This peak broadening is a direct indicator of increased lattice strain and a decrease in the coherent scattering domain size, which can be associated with grain refinement and the accumulation of crystal defects like dislocations [27,28]. It is noteworthy that the overlapping of broadened α-phase peaks may obscure the identification of the β phase. The XRD results confirm that SMAT significantly alters the near-surface phase integrity and induces severe lattice strain in the dominant α phase. Direct evidence and quantification of grain refinement and microstructural evolution are provided by the EBSD and SEM analyses. The attenuation and eventual disappearance of the β-phase diffraction peaks with increasing SMAT duration can be attributed to several interrelated factors. The primary cause is believed to be the extreme grain refinement and the introduction of severe lattice strain within the β phase, leading to significant peak broadening that renders its signal undetectable amidst the background and the broadened α-phase peaks. Additionally, a deformation-induced phase transformation from the metastable β phase to α’ martensite under the high-strain-rate conditions of SMAT could also contribute to the reduction in β-phase volume fraction. Definitive confirmation of such a transformation would require further analysis using transmission electron microscopy (TEM).

### 3.2. Microstructure and Mechanical Properties of SMAT-Treated TC4

**Figure 6:**
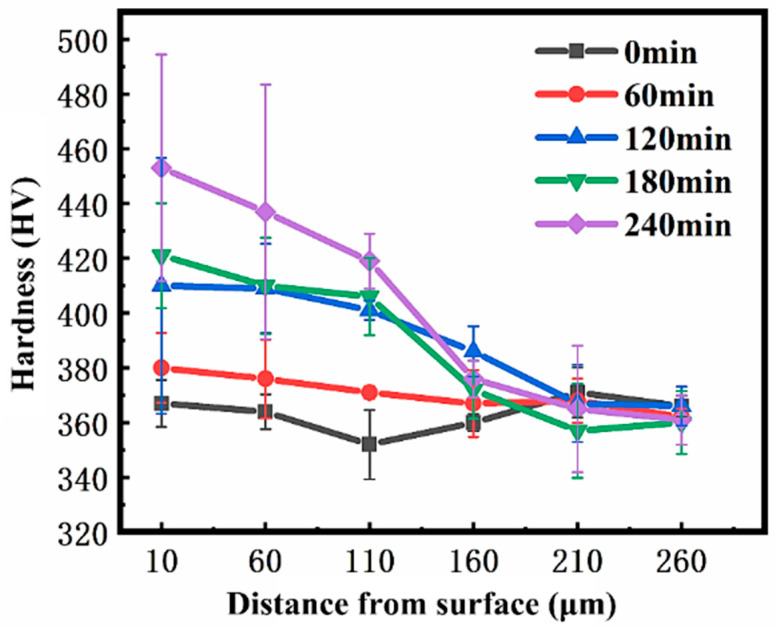
The hardness curves of SMAT samples at different times in the direction of surface depth.

The Vickers hardness of the surface and cross-section of the sample after SMAT was tested and analyzed. Because the Vickers hardness is more accurate when the diagonal length of the diamond indentation exceeds 20 μm, 100 g load is selected for the test, and the load is kept for 10 s. Measurements were performed along a straight line on the cross-section, covering the original microstructure matrix and different depths from the surface (10 μm, 60 μm, 110 μm, 160 μm, 210 μm, 260 μm) are measured. The average Vickers hardness of the original structure was 364 by 5-point measurement. Table 3 lists the results of Vickers hardness measured on the cross-section of the sample after different SMAT times. The results show that in the depth direction, with the increase in SMAT time, the maximum hardness of TC4 alloy gradually increases: it reaches 380 after 60 min, 410 after 120 min, 421 after 180 min, and 453 after 240 min. Figure 6 shows that the hardness value of TC4 alloy after SMAT for 60 min, 120 min, 180 min, and 240 min increases with the increase in SMAT time in the range of 0–260 μm from the treated surface, and reaches the maximum hardness at the nearest place from the surface. As the distance from the surface increases, the hardness gradually decreases. In the depth range of 210~260μm, the cross-section hardness of the samples with different treatment times gradually tends to be consistent, close to the matrix hardness, indicating that the hardening phenomenon is weakened in this depth range, and the effect of SMAT on the material basically disappears. It is worth noting that in the range of 160~260 μm, the hardness of the 120 min sample is slightly higher than that of the 180 min sample. This may be attributed to the more effective propagation of plastic deformation into the subsurface region due to the extended SMAT duration. The increase in SMAT time enhances the kinetic energy and frequency of impacts, which can promote deeper dislocation activity and grain refinement. Although the difference becomes smaller with depth, the 120 min sample still retains a slightly stronger hardening effect, suggesting a deeper influence of SMAT at this duration. The larger standard deviation in hardness values within the top ~60 µm layer, as indicated by the error bars in Figure 6, can be attributed to the inherent microstructural inhomogeneity and the gradient distribution of plastic strain in the severe plastically deformed surface layer, as confirmed by the EBSD and KAM analyses. The increased hardness and altered wear properties are a direct consequence of the high dislocation density and severe lattice distortion (i.e., the stored strain energy) revealed by our KAM and XRD analyses.

**Figure 7:**
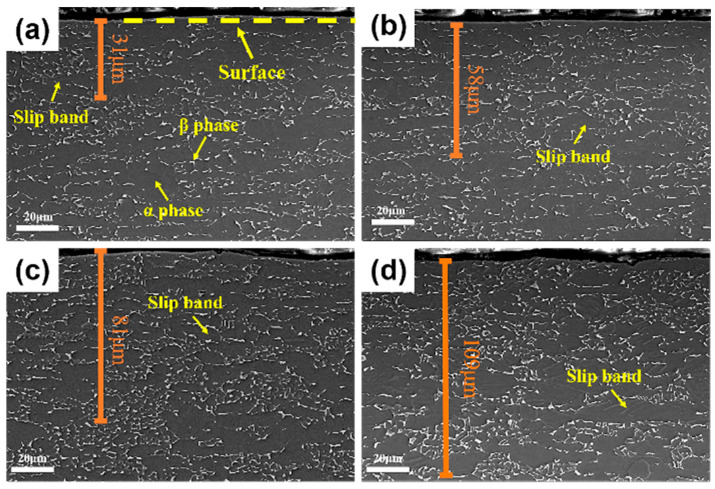
The depth diagram of slip band in TC4 section after different times of SMAT. (**a**) 60 min, (**b**) 120 min, (**c**) 180 min, (**d**) 240 min.

The cross-sectional SEM images in Figure 7 reveal the evolution of plastic deformation in TC4 alloy with increasing SMAT time. A key observation is the progressive increase in the depth of slip bands, predominantly within the α phase, from approximately 31 μm at 60 min to 109 μm at 240 min. This contrasts sharply with the β phase, which maintains its original morphology without significant deformation even after prolonged SMAT. This disparity is attributed to the enrichment of V in the β phase, which strengthens it and markedly reduces its plastic deformation capability compared to the α phase [27,28]. Consequently, the α phase undergoes severe plastic deformation via slip, while the β phase remains largely unaffected.

**Figure 8:**
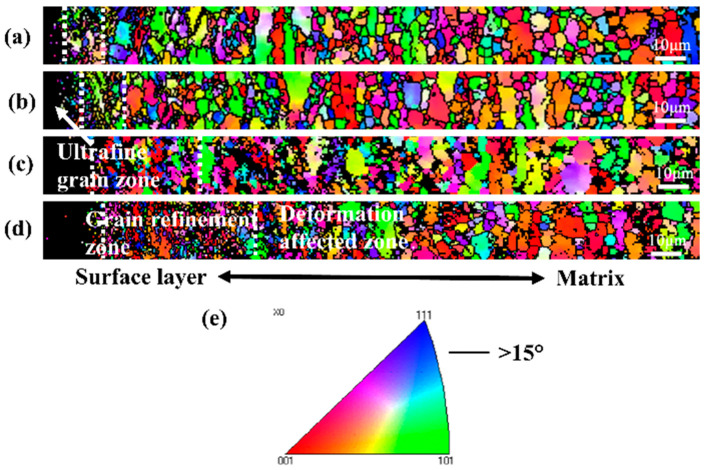
EBSD grain orientation diagram of TC4 surface cross-section structure after SMAT for different times. (**a**) 60 min, (**b**) 120 min, (**c**) 180 min, (**d**) 240 min, (**e**) reverse pole figure.

Figure 8 shows the EBSD grain orientation map of the cross-section microstructure of TC4 alloy after different SMAT times. It can be seen from the figure that at 60 min of SMAT, the black uncalibrated area first appeared on the surface of the TC4 alloy. The grains in this area have been refined, and the size is smaller than the EBSD detection accuracy, so it cannot be calibrated, showing a black uncalibrated area. On the right side of the region, there are obvious fine grains, and from the surface layer to the matrix direction, the grains gradually increase, and finally are consistent with the matrix grains. From the EBSD images of SMAT 120 min, SMAT 180 min, and SMAT 240 min, it can be seen that with the extension of SMAT time, the gradient grain refinement from the surface layer to the matrix is shown in the surface structure at all treatment times. In this paper, we divide the gradient grain refinement zone into ultrafine grain zone, grain refinement zone, deformation affected zone, and matrix zone. Since the grain size of the ultrafine-grained region has reached or is close to the nanometer level, it is difficult to calibrate in the EBSD image, so it is presented as a black uncalibrated region. The right side of the black zone is the grain refinement zone, and the further right side is the deformation affected zone and the matrix zone. However, since the deformation-affected zone has little effect on the grain size, these two regions are difficult to distinguish on the grain orientation map. In general, with the increase in SMAT time, the thickness of the grain refinement zone gradually increases.

**Figure 9:**
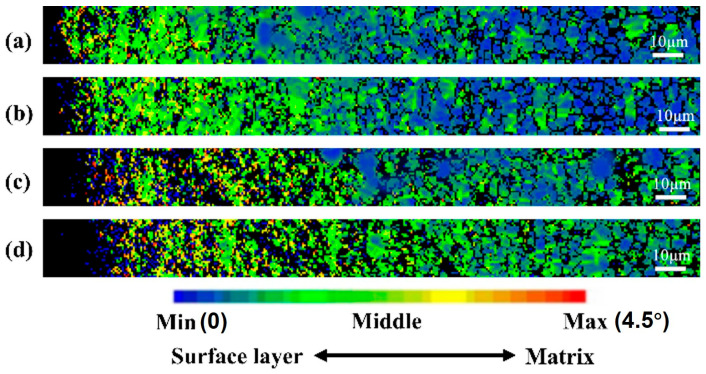
The local misorientation spread maps of TC4 cross-section microstructure after SMAT at different times. (**a**) 60 min, (**b**) 120 min, (**c**) 180 min, (**d**) 240 min.

Figure 9 shows the Kernel Average Misorientation (KAM) distribution maps of the cross-sectional microstructure of TC4 alloy after SMAT for different durations. KAM values reflect the degree of local crystal orientation variation and are closely associated with plastic strain, dislocation density, and lattice distortion, making them a key indicator for analyzing microstructural deformation and internal strain accumulation. At 60 min of SMAT (Figure 9a), regions with low KAM values (blue–green) dominate, and only sparse high-KAM zones (yellow to red) are observed within approximately 10 μm of the surface, indicating limited plastic deformation and a relatively low dislocation density. As the treatment duration increases to 120 min (Figure 9b), the high-KAM region near the surface becomes more prominent and penetrates deeper, extending to a depth of around 15–20 μm, suggesting intensified plastic deformation and increased dislocation accumulation. At 180 min (Figure 9c), red high-KAM zones expand further toward the substrate, reaching a depth of 25–30 μm, which signifies a more severe degree of lattice distortion and internal strain. After 240 min of SMAT (Figure 9d), the high-KAM regions become more concentrated and continue to grow inward, with maximum misorientation values markedly increased. This implies that prolonged SMAT leads to extensive dislocation accumulation and crystal reorientation, resulting in intense plastic deformation. Overall, with increasing SMAT duration, the local misorientation gradually intensifies and extends from the surface toward the substrate, reflecting a progressive enhancement in surface plastic strain and a deepening of the deformation-affected layer. This trend demonstrates the potential of SMAT in introducing gradient structures in the surface region and improving the mechanical performance of the material.

**Figure 10:**
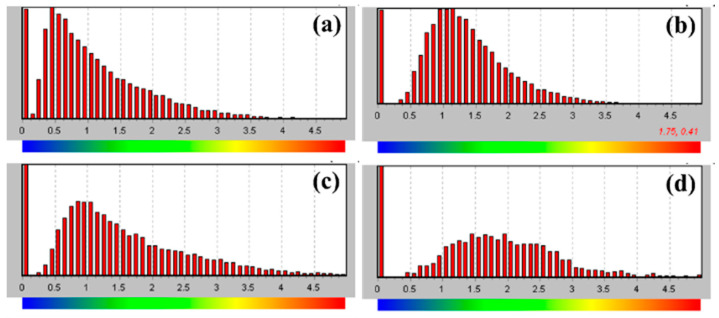
Local orientation difference statistics of TC4 surface cross-section structure after SMAT for different times. (**a**) 60 min, (**b**) 120 min, (**c**) 180 min, (**d**) 240 min.

Figure 10 shows the local misorientation statistical diagram corresponding to the strain distribution diagram in Figure 9, the x-axis represents the Kernel Average Misorientation (KAM) angle in degrees, which reflects the degree of local lattice distortion within individual grains. The y-axis represents the frequency (i.e., the number of pixels) corresponding to each misorientation angle, based on EBSD mapping over the top 200 μm of the sample cross-section. From the map, it can be seen that from SMAT 60 min to 240 min, the proportion of areas with a local orientation difference of 0° is always high, which indicates that under each treatment, there are more unaffected areas in the surface 200 μm range. With the extension of SMAT time, the peak value of local misorientation gradually moves from the left to the right, indicating that the angle of local misorientation gradually increases.

The significant grain refinement and increase in surface hardness observed in this study are consistent with the well-documented effects of SMAT on metallic materials [13]. However, the systematic quantification of the gradient layer thickness and its direct correlation with treatment time provides a more detailed roadmap for process optimization in TC4 alloy compared to earlier studies [16].

### 3.3. Effect of Different SMAT Time on Fretting Wear Properties of TC4 Alloy

**Figure 11:**
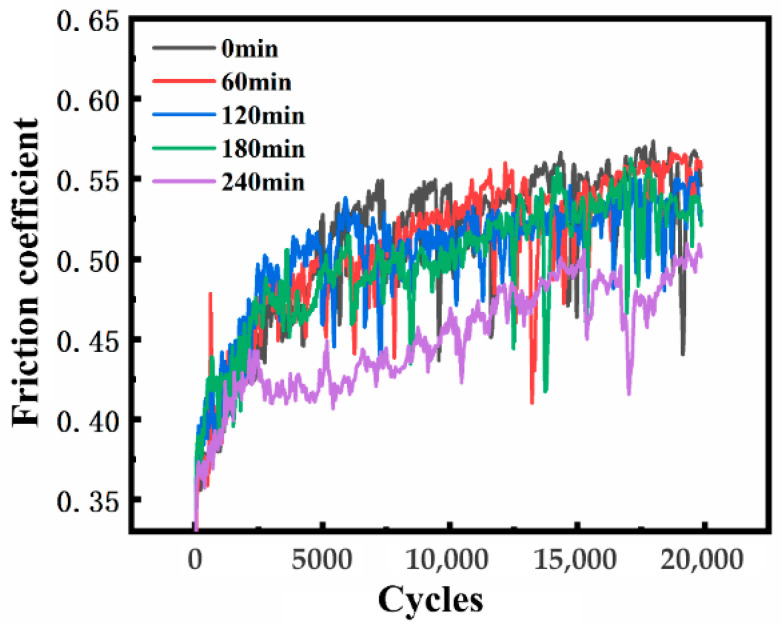
The friction coefficient curves of fretting wear of SMAT samples at different times after 20,000 cycles were plotted.

Figure 11 shows the friction coefficient curves of SMAT 0 min, 60 min, 120 min, 180 min, and 240 min samples in the fretting wear of 20,000 cycles. It can be seen from the diagram that with the increase in fretting wear cycles, the friction coefficient of the five samples experienced an upward stage before 10,000 cycles, and then tended to fluctuate and stabilize. At about 20 to 10,000 cycles, the wear process enters the transition stage from two-body to three-body. At this stage, as the wear progresses, the wear debris between the contact surfaces continues to form and gradually accumulates into a third body, similar to the ball effect, which significantly reduces the friction coefficient. However, the wear debris generation rate is greater than its overflow rate, resulting in a small range of friction coefficient tending to be stable and of slow growth, from 0.15 to more than 0.5. After about 10,000 cycles, the surface particles continue to peel off, and the wear debris is oxidized and fragmented under the action of fretting extrusion. The generation rate of wear debris is basically equal to the overflow rate, maintaining a dynamic balance, and the friction coefficient is maintained at about 0.55 and the change is small.

**Figure 12:**
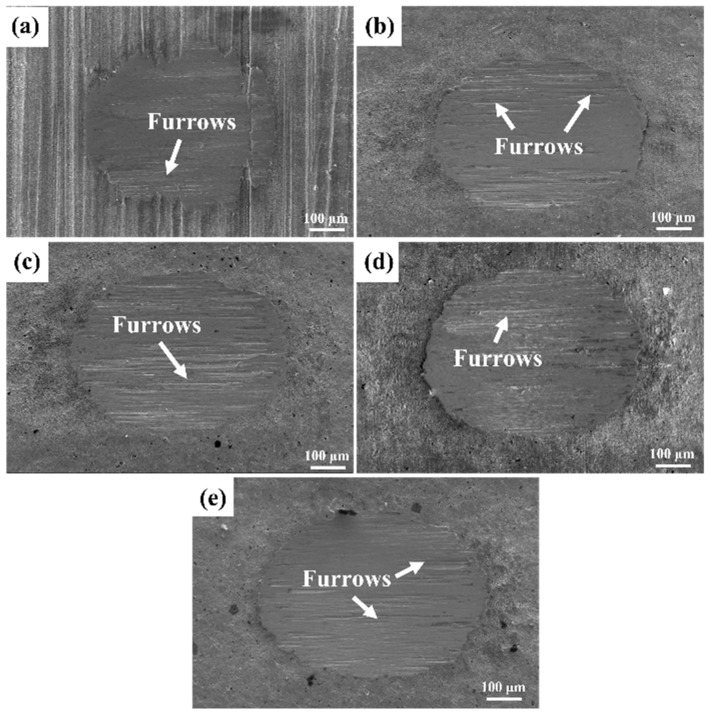
The SEM images of the fretting wear surface morphology of the SMAT after different times. (**a**) 0 min, (**b**) 60 min, (**c**) 120 min, (**d**) 180 min, (**e**) 240 min.

Figure 12 shows the SEM images of the surface morphology of SMAT samples with different treatment times after fretting wear for 20,000 cycles under 2 N load. It can be seen from the figure that with the increase in SMAT time from 0 min to 240 min, the wear scar generally presents a nearly elliptical shape. No matter how the treatment time changes, the deep furrows showing “white” likely being “oxide-rich tribolayers” under the electron microscope are distributed in all parts of the wear marks, indicating that the fretting wear mode before and after SMAT is the complete slip zone mode under 2 N load. SMAT did not significantly change the wear mode. In the surface contour map, the edge of the wear scar is significantly higher than that of the wear scar and the substrate surface, which is mainly due to the plastic deformation of the material at the edge of the wear scar under the extrusion of the grinding ball, resulting in material transfer. In addition, the adhesion of wear debris on the edge is also the reason why the edge of the wear scar is higher than that of the substrate. When the wear debris accumulates at the edge of the wear scar, a small gap may be formed on the surface, which may lead to gap corrosion, resulting in accelerated corrosion rate, and even galvanic corrosion may occur due to the difference in material potential.

**Figure 13:**
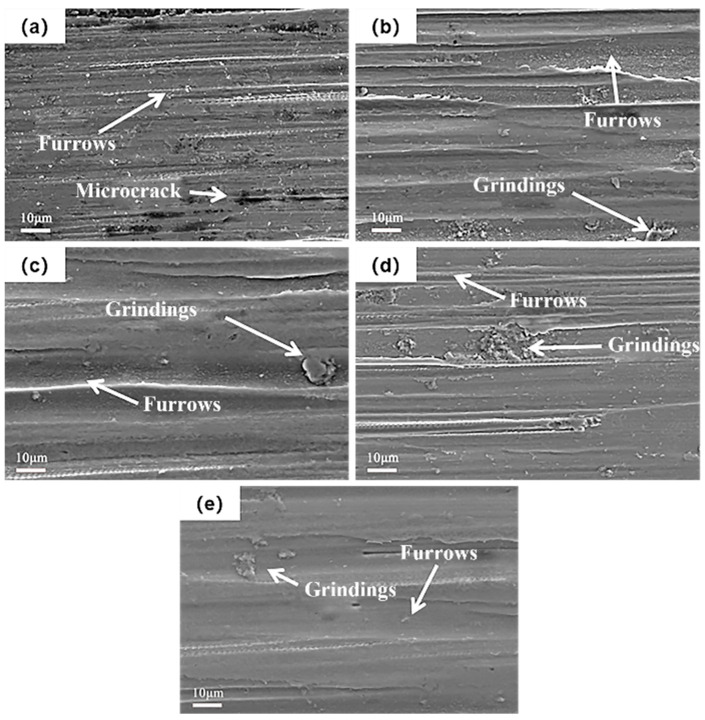
The micro-morphology of wear scar after fretting wear of SMAT at different times. (**a**) 0 min, (**b**) 60 min, (**c**) 120 min, (**d**) 180 min, (**e**) 240 min.

Figure 13 shows the micro-morphology of the wear scar after fretting wear of SMAT at different treatment times. It can be seen from the figure that the fretting wear mode of the original TC4 alloy under the experimental conditions is jointly affected by adhesive wear, abrasive wear, and delamination wear. After SMAT, the wear mode is mainly transformed into adhesive wear, supplemented by abrasive wear. With the increase in SMAT time, the proportion of delamination wear gradually decreases, while the influence of adhesive wear gradually increases. According to the literature, the TTS layer generated during fretting wear is usually composed of equiaxed nanocrystals, which play a key role in reducing the friction coefficient and maintaining the steady state of fretting wear [29,30]. The wear resistance of the samples after SMAT is improved, mainly due to the nanocrystalline layer formed in advance on the surface of TC4 alloy, which significantly reduces the friction coefficient and promotes the fretting wear to accelerate into the three-body wear stage of the nanocrystalline layer.

The reduction in delamination wear and the shift towards adhesive wear dominant mechanism after SMAT can be attributed to the refined surface microstructure. The pre-formed nanocrystalline layer in our SMAT samples likely functions similarly to the tribologically transformed structure (TTS) layers reported to stabilize fretting wear in other systems [29]. This pre-existing layer facilitates an earlier transition to a stable three-body wear regime, thereby reducing wear volume and rate, a finding that aligns with the wear resistance improvement reported by Kumar et al. [16] but further quantifies the effect of processing duration. The reduction in delamination wear is attributed to the refined grain structure, which inhibits the initiation and propagation of subsurface cracks. The shift towards adhesive wear is discussed in the context of the smoother, nanocrystalline surface facilitating more intimate contact, while the stable tribolayer prevents severe material transfer.

**Figure 14:**
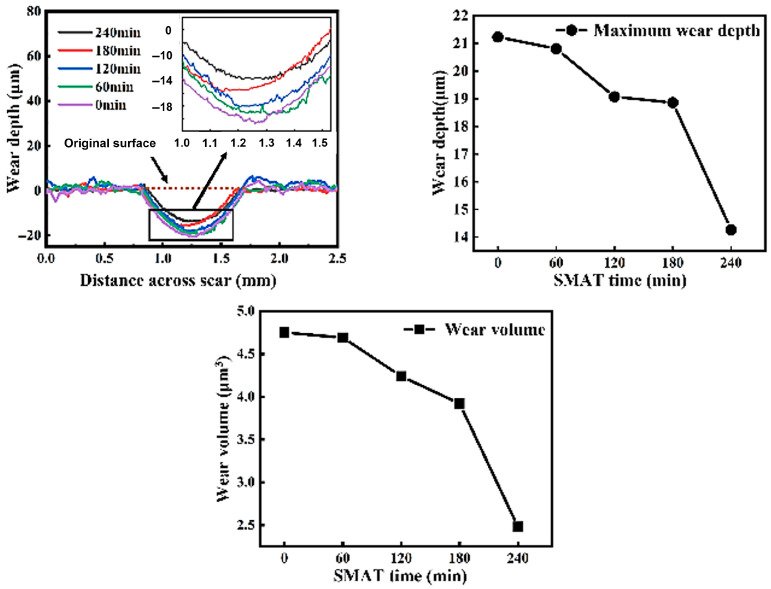
The wear scar cross-section profile, wear depth, and wear volume of the sample after SMAT for different times.

Figure 14 shows that the wear depth, wear volume, and wear rate of the SMAT-treated samples are significantly reduced compared with the untreated matrix samples. As quantitatively summarized in Figure 14, the wear depth decreased from 21.23 μm for the untreated sample to 14.27 μm after 240 min of SMAT, representing a 32.8% reduction. Similarly, the wear volume and wear rate were reduced by nearly half, from 4.75 × 10^6^ μm^3^ and 2.375 × 10^3^ μm^3^/s to 2.48 × 10^6^ μm^3^ and 1.24 × 10^3^ μm^3^/s, respectively, corresponding to a ~48% improvement.

The SMAT-induced nanocrystalline surface enhances the formation of a more continuous and adherent passive film due to the high density of grain boundaries acting as rapid diffusion paths for oxygen. During fretting, this layer is repeatedly removed (wear) and reformed (corrosion). The refined microstructure of the SMAT-treated surface likely facilitates faster repassivation and improves the stability of the tribolayer, thereby reducing the damage from synergistic wear–corrosion. This mechanism contributes to the overall improvement in wear resistance.

## 4. Conclusions

In this paper, white light interferometer, EBSD, SEM, XRD, and microhardness measurements were used to systematically characterize the surface morphology, cross-section structure, and fretting wear properties of the matrix without SMAT and the TC4 alloy treated with SMAT for different times (60 min, 120 min, 180 min, 240 min). The trend of surface morphology, interface structure, and wear properties with treatment time was studied, and the following conclusions were drawn:

1. SMAT changes the surface morphology and roughness of TC4 alloy. With the increase in SMAT time, the plow grooves on the original surface disappear and are replaced by shallow pits, and the surface roughness (Ra) increases from 366 nm to 1133 nm.
2. SMAT leads to severe plastic deformation of TC4 alloy and significantly refines the grain size. With the increase in SMAT time, the strain significantly increases and the deformation depth increases to 200 μm. The surface grains are obviously refined to nanometer size.
3. SMAT significantly improves the fretting wear resistance of TC4 alloy. After 240 min of treatment, the wear depth decreased from 21.23 μm to 14.27 μm (a 32.8% reduction), while the wear volume and wear rate were reduced from 4.75 × 10^6^ μm^3^ and 2.375 × 10^3^ μm^3^/s to 2.48 × 10^6^ μm^3^ and 1.24 × 10^3^ μm^3^/s, respectively (representing a ~48% improvement.

## Figures and Tables

**Table 1:** Parameters of SNC-1 surface nanocrystallization testing machine.

Input Voltage	Power	Operating Frequency	Working Vessel Vacuum	Processing Size
AC 380 V	3 kw	~50 Hz	>5 Pa (−0.08 MPa)	100 mm × 100 mm

**Table 2:** Composition of artificial seawater (according to ASTM D1141-98) [26].

Compound	Chemical Formula	Concentration (g/L)
Sodium Chloride	NaCl	24.53
Magnesium Chloride	MgCl_2_	5.20
Sodium Sulfate	Na_2_SO_4_	4.09
Calcium Chloride	CaCl_2_	1.16
Potassium Chloride	KCl	0.695
Sodium Bicarbonate	NaHCO_3_	0.201
Potassium Bromide	KBr	0.101
Boric Acid	H_3_BO_3_	0.027
Strontium Chloride	SrCl_2_	0.025
Sodium Fluoride	NaF	0.003

**Table 3:** The hardness values of the samples at different depths from the surface at different SMAT times.

Depth from Surface (μm)	Hardness (HV)				
	0 min	60 min	120 min	180 min	240 min
10	367	380	410	421	453
60	364	376	409	410	437
110	352	371	401	406	419
160	360	367	386	372	376
210	371	368	367	357	365
260	366	362	366	360	361

## Data Availability

The original contributions presented in this study are included in the article. Further inquiries can be directed to the corresponding authors.
